# Incidence and risk factors of acute kidney injury in patients with malignant tumors: a systematic review and meta-analysis

**DOI:** 10.1186/s12885-023-11561-3

**Published:** 2023-11-17

**Authors:** Wang Can, Li Rong, Liu Lixia

**Affiliations:** Department of Critical Care Medicine, the Fourth Hospital of Hebei Medical University, Shi Jiazhuang, China

**Keywords:** Tumor, Acute kidney injury, Intensive care, Risk factors, Systematic review, Meta-analysis

## Abstract

**Background:**

There are significant differences in the incidence and risk factors of tumor patients, and there is no relevant statistical data. Therefore, this study aims to clarify the incidence and risk factors of acute kidney injury (AKI) in malignant tumor patients and compare critically ill patients with non-critically ill patients.

**Methods:**

Relevant literature on the occurrence of AKI in malignant tumors was retrieved from databases. Two authors independently screened and evaluated the eligibility and quality of the literature and extracted the data. The Stata 12.0 software was used for meta-analysis.

**Results:**

A total of 3922 articles were initially retrieved, and 24 articles were finally included, 8 of which were about critically ill malignant tumor patients, and 16 were about malignant tumor patients. Among the 4107 patients included in the 8 studies on critically ill malignant tumors, 1932 developed AKI, with an incidence rate of 52% (95%CI 34–70%, I2 = 99%). The risk factors for AKI in critically ill malignant tumor patients were sepsis and hypovolemia, which were different from those in non-critically ill patients. Among the 292,874 patients included in the 16 studies on malignant tumors, 51,211 developed AKI, and the combined incidence rate was 24% (95%CI 17–30%, I2 = 100%). The risk factors for AKI in critical malignant tumor patients were sepsis and hypovolemia.

**Conclusion:**

This meta-analysis shows that the incidence of AKI in critically ill malignant tumor patients is consistent with that in other critically ill patients, and independent risk factors are sepsis and hypovolemia. The incidence of AKI in malignant tumor patients is higher than that in other patients, and tumor is a risk factor for AKI. This study has been registered in INPLASY (INPLASY202320079),Registered February 18,2023.

**Supplementary Information:**

The online version contains supplementary material available at 10.1186/s12885-023-11561-3.

## Introduce

Acute kidney injury (AKI) is a syndrome characterized by a rapid loss of renal excretory function, typically diagnosed by the accumulation of nitrogenous metabolic end products (urea and creatinine) and/or reduced urine output. It represents a clinical manifestation of several diseases that acutely affect the kidney. AKI is common in hospitalized patients, particularly in critically ill patients, and is most commonly secondary to extrarenal events. The mechanism by which these events lead to AKI is controversial [1]. In recent years, the incidence of cancer has been increasing, and with the emergence of new methods for treating various tumors and the prolongation of survival times for cancer patients, the chances of renal involvement in cancer patients have significantly increased [2]. A meta-analysis reported the global incidence of AKI: according to the KDIGO definition, 1 in 5 adults worldwide experience AKI during hospitalization [3]. To date, there has been no meta-analysis of the incidence of AKI in cancer patients. Therefore, this study takes a comprehensive approach to review relevant literature and perform a systematic review and meta-analysis of the incidence and risk factors of AKI in cancer patients.

### Methods

Prior to this study, a protocol was developed that described the objectives, search strategy, and analysis plan for this systematic review and meta-analysis. This study was conducted in accordance with the Preferred Reporting Items for Systematic Reviews and Meta-analyses (PRISMA) [4] project. The protocol for this systematic review was registered on INPLASY (Unique ID number) and is available in full on inplasy.com (https://doi.org/10.37766/inplasy202320079).

## Data sources

Two reviewers systematically searched PubMed, Embase, Cochrane, CNKI, and CBM databases for relevant literature on the occurrence of acute kidney injury (AKI) in malignancies from inception to December 19, 2022. The search terms and free words were established based on official medical dictionaries and extensive reading of the literature. The search terms included "Neoplasms", "Acute Kidney Injury", and "risk", and the free words included "tumor", "neoplasm", "tumors", "neoplasia", "neoplasias", "cancer", "malignancy", "AKI", "acute renal failure", "factors", and "cohort". (The detailed "PubMed" search syntax is provided in Appendix Table: ("Neoplasms"[MeSH Terms] OR ("tumor"[Title/Abstract] OR "neoplasm"[Title/Abstract] OR "tumors"[Title/Abstract] OR "neoplasia"[Title/Abstract] OR "neoplasias"[Title/Abstract] OR "cancer"[Title/Abstract] OR "malignancy"[Title/Abstract])) AND ("Acute Kidney Injury"[MeSH Terms] OR ("AKI"[Title/Abstract] OR "acute renal failure"[Title/Abstract] OR "Acute Kidney Injury"[Title/Abstract])) AND ("risk"[Title/Abstract] OR "risk"[MeSH Terms] OR "factors"[Title/Abstract] OR "cohort"[Title/Abstract])).

## Study selection and data extraction

### The inclusion and exclusion criteria of this study were developed according to the PICOS principle.

#### Inclusion criteria

(1) The main diagnostic criteria for AKI were the serum creatinine (Scr) and urine output (UO) standards proposed by the Kidney Disease Improving Global Outcomes (KDIGO), the RIFLE criteria, and the standards proposed by Bellomo et al. (2) Study subjects: cancer patients, regardless of cancer type, aged ≥ 18 years, divided into case group (AKI group) and control group (non-AKI group) based on whether AKI occurred. (3) Study content: Each study reported the incidence of AKI or risk factors for acute kidney injury. (4) Study type: Case–control study, cohort study, or studies related to the incidence or risk factors of AKI in cancer patients. (5) Outcome indicators: The original literature provided the incidence and risk factors of AKI in cancer patients.

#### Exclusion criteria

(1) inability to provide original data; (2) duplicate publication of the study; (3) case reports or animal experiments; (4) incomplete or non-compliant data with the extraction criteria; (5) less than 50 subjects in the study.

#### Data extraction

Two reviewers independently conducted data extraction through full-text reading of the included articles. The reviewers developed a preliminary data extraction form during the screening process and gradually refined the content of the data extraction form during the full-text reading process. Data extracted included the first author, year of publication, study start and end time, sample size, tumor type, age, gender, AKI diagnostic criteria, AKI incidence rate, and AKI risk factors. If there was a control group, we also extracted exposure factors such as age, gender, body mass index (BMI), and acute physiology and chronic health evaluation II (APACHE II) score.

### Assessment of methodological quality

This study is an observational study, and all included studies are cohort studies. The Newcastle–Ottawa Scale (NOS) was used for quality assessment, including three aspects: selection of study subjects, comparability between groups, and evaluation of outcomes. The more items that are met, the higher the quality of the study.

### Data statistics and analysis

After a large amount of preliminary reading of systematic reviews on this study, all data that can be quantitatively analyzed were synthesized using the stata software. The incidence rate and its 95% confidence interval (CI) were generated by the software and presented in a forest plot. The heterogeneity between studies was evaluated using the I2 statistic. When I2 ≤ 50%, it indicates that there is no statistical heterogeneity between the studies, and a fixed-effect model (FE) is used for meta-analysis. When I2 > 50%, it indicates that there is statistical heterogeneity between the studies, and a random-effect model (RE) is used for analysis. When significant heterogeneity is found in the main outcome indicators, sensitivity analysis is used to identify the reason for the heterogeneity. A P-value < 0.05 is considered statistically significant. Subgroup analysis of AKI incidence rate was performed based on tumor type. A funnel plot was used to assess publication bias. If the distribution of the funnel plot is symmetric, there is no publication bias; otherwise, publication bias exists.

## Results

### Study characteristics

**Fig. 1:**
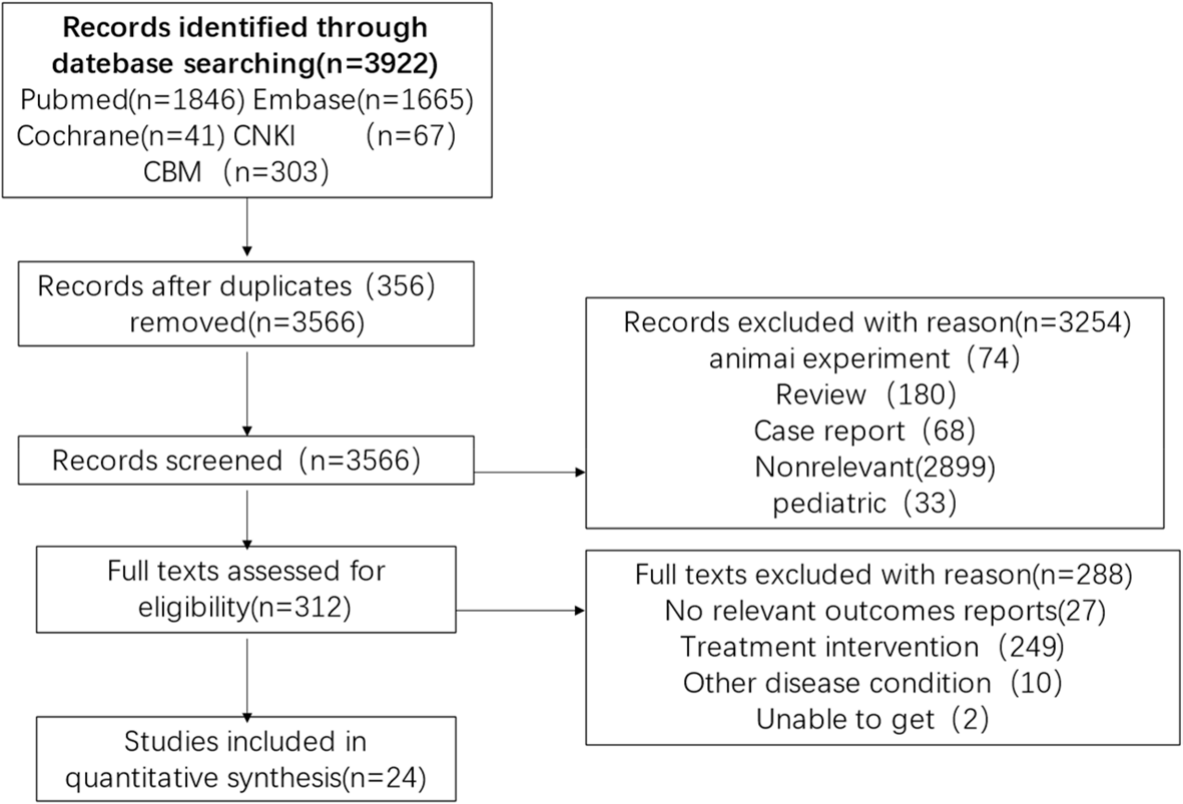
Literature screening process and results

The PRISMA flow diagram is shown in Fig. 1. A total of 3,922 articles were identified through the search strategy, with 356 duplicate studies excluded. Two reviewers independently screened titles and abstracts of 3,566 articles, and excluded 3,254 articles that were not relevant to the study topic. After reading the full text of 312 studies, 280 articles were excluded due to lack of relevant data, and finally 24 studies were included, including 16 studies of cancer patients and 8 studies of critically ill cancer patients, all reporting the incidence of AKI in patients. Tables 1 present detailed characteristics of the included studies in critically ill cancer patients and cancer patients, respectively.

**Table 1:** Characteristics of the literature on patients with critical malignancies included in the study

	Study start and end dates	Sample size	Tumor types	Age	Male	AKI diagnostic criteria	Total AKI incidence	Solid tumor AKI Incidence	Incidence of AKI in hematologic tumors	AKI risk factors
(Córdova-Sánchez, Herrera-Gómez et al. 2016) [5]	2013.1 -2015.3	389	Solid tumors (n = 280) Hematologic tumors (n = 109)	50 IQR 35–61	180	KDIGO	69.4	65	80	–
(Córdova-Sánchez, Ruiz-García et al. 2019) [6]	2014.4–2015.7	96	Solid tumors (n = 67) Hematologic tumors (n = 29)	–	41	KDIGO	63	55.2	79.3	SOFA score, low hemoglobin
(Darmon, Vincent et al. 2015) [7]	2010 -2012	1009	Hematologic tumors (n = 1009)	–	–	AKIN	66.5	–	66.5	Age, SOFA score, history of hypertension, tumor lysis syndrome, exposure to nephrotoxic drugs, and myeloma
(Kemlin, Biard et al. 2018) [8]	2011.1 -2015.12	204	Solid tumors (n = 204)	64 (53–70)	118	KDIGO	59	59	–-	Sepsis, hypovolemia and outflow tract obstruction, Simplified acute physiological score, abdominopelvic cancer, nephrotoxic chemotherapy within the last 3 months
(Libório, Abreu et al. 2011) [9]	2006.5 -2008.6	288	Solid tumors (n = 258) Hematologic tumors (n = 30)	58.8 ± 17.4	150	RIFLE	54.2	55.8	40	Age, lower hemoglobin, poorer renal function (APACHE II, SOFA and SAPS II) scores, high creatinine on admission
(Seylanova, Crichton et al. 2020) [10]	2004.1 -2012.7	429	Solid tumors (n = 335) Hematologic tumors (n = 94)	–	–	KDIGO	60	44.8	70.2	–
(Soares, Lobo et al. 2010) [11]	2007.8.1 -9.30	717	Solid tumors (n = 667) Hematologic tumors (n = 50)	–	–	RIFLE	12	11	26	–
(Soares, Salluh et al. 2006) [12]	2000.5 -2004.12	975	–	60.9 ± 15.9	–	Bellomo	32	–	–	–
(Cheng, Nie et al. 2019) [13]	2013. 1. 1- 2015.12.31	136,756	–	–	–	KDIGO	0.075	–	–	Age, elevated baseline blood creatinine, shock and urinary tract obstruction
(Cho, Kang et al. 2019) [14]	2004.1.1–2013.12.31	3,202	Solid tumors (n = 3202)	63.8 ± 10.3	31.4%	KDIGO	0.557	0.557	–	–
(Christiansen, Johansen et al. 2011) [15]	1999.1.1–2006.12.31	37,267	Solid tumors (n = 34,491) Hematologic tumors (n = 2776)	–	21,387	RIFLE	0.258	–	–	–
(Kang, Park et al. 2019) [16]	2004. 1—2013. 12	68,036	–	56.9 ± 13.0	34,414 (50.6)	KDIGO	0.338	–	–	Age, male, diabetes, hypertension, low initial renal function, low serum albumin and serum hemoglobin
(Lahoti, Kantarjian et al. 2010) [17]	1999–2007	537	Hematologic tumors (n = 537)	56 (17–84)	281	RIFLE	0.36	–	0.36	Age, mechanical ventilation, use of vancomycin, diuretics, amphotericin B lipid preparations, vasopressants, leukopenia, hypoproteinemia, chemotherapy
(Li, Wang et al. 2021) [18]	2014.4 -2019.3	3127	Solid tumors (n = 2906) Hematologic tumors (n = 221)	–	–	–	0.211			–
(May, Barreto et al. 2021) [19]	2005—2017	1069	Hematologic tumors (n = 1069)	67 (54–76)	624 (58)	KDIGO		–		–
(Abdeljaleel et al. 2022) [20]	2016.1–2018.1	204	–	54.7 ± 12.9	48.4%	RIFLE	0.069	–	–	Congestive heart failure, chronic kidney disease, hypercalcemia
(Nowshad, Alam et al. 2010) [21]	2006. 5. 1 – 2006. 7. 31	5013	–	54 ± 18	52%	Absolute increase in serum creatinine ≥ 0.3 mg/dl	0.28	–	–	Chemotherapy, antidiabetic and antibiotic medications, hyperglycemia, admission through an emergency center or to an ICU, admission to a hospital service group related to or cancer type admission for stem cell transplantation or malignant hematologic disease
(Rainone, Merlino et al. 2013) [22]	–	170	Hematologic tumors (n = 170)	66 ± 11.1	55%	KDIGO	0.45	–	0.45	Hypercalcemia, hyperphosphatemia, proteinuria, low hemoglobin
(Salahudeen, Doshi et al. 2013) [23]	2006年3个月	3558	Solid tumors (n = 2704) Hematologic tumors (n = 854)	56 ± 17	52%	RIFLE	0.12			Diabetes, chemotherapy, intravenous contrast, hyponatremia, antibiotics
(Shen, Wang et al. 2020) [24]	2014.10.1 -2015.9.30	16,082	–	61(51, 68)	10,388 (65.4%)	KDIGO	0.148	–	–	Lower serum magnesium, age, higher WBC, lower ALB and HGB
He Xiaoqin, YangMin et al. [25]	2011.1.1–2020.12.31	15 937	–	–	–	KDIGO	0.0426	–	–	–
Li Yuejia et al. [26]	2017.1.1–2019.12.31	197	Hematological system (n = 197)	–	–	KDIGO	0.167	–	0.167	Hyperuricemia, anemia, chemotherapy, hypertension
Liu Shuiying, et al. [27]	2019.1–12	1 658	–	61.23 ± 16.14	1.05∶1	KDIGO	0.136	–	–	Age, tumor metastasis, CKD, hypoalbuminemia, hyperuricemia, prenephrotic factors, infection, urinary tract obstruction, chemotherapy, use of NSAID, diuretics
Peng Zhangjing [28]	2017.1–2019.12	161	Hematological system (n = 161)	62.02 ± 11.09	92	KDIGO	0.1242	–	0.1242	High blood phosphorus, low hemoglobin

A total of 4,105 critically ill cancer patients and 292,874 cancer patients were included for quantitative synthesis. The Newcastle–Ottawa Scale (NOS) was used to assess the quality of the included studies, all of which were observational studies. Table 2 shows the NOS scores of the included studies.

**Table 2:** Modify: Newcastle–Ottawa Scale (NOS) quality assessment

Study	Selection				Comparability		Outcome			
	Representative-ness	Selection of non-exposed	Ascertainment of exposure	Outcome not present at start	Comparability on most important factors	Comparability on other risk factors	Assessment of outcome	Long enough follow-up	Adequacy (completeness) of follow-up	Score
(Córdova-Sánchez, Herrera-Gómez et al. 2016) [5]	*	*	*	-	*	*	*	*	*	********
(Córdova-Sánchez, Ruiz-García et al. 2019) [6]	*	*	*	-	*	*	*	*	*	********
(Darmon, Vincent et al. 2015) [7]	*	*	*	-	*	*	*	*	*	********
(Kemlin, Biard et al. 2018) [8]	*	*	*	-	*	*	*	*	*	********
(Libório, Abreu et al. 2011) [9]	*	*	*	-	*	*	*	*	*	********
(Seylanova, Crichton et al. 2020) [10]	*	-	*	-	-	-	*	*	*	*****
(Soares, Lobo et al. 2010) [11]	*	-	*	-	-	-	*	*	*	*****
(Soares, Salluh et al. 2006) [12]	*	-	*	-	-	-	*	*	*	*****
(Cheng, Nie et al. 2019) [13]	*	*	*	-	*	*	*	*	*	********
(Cho, Kang et al. 2019) [14]	*	-	*	-	-	-	*	*	*	*****
(Christiansen, Johansen et al. 2011) [15]	*	-	*	-	-	-	*	*	*	*****
(Kang, Park et al. 2019) [16]	*	-	*	-	-	-	*	*	*	*****
(Lahoti, Kantarjian et al. 2010) [17]	*	*	*	-	*	*	*	*	*	********
(Li, Wang et al. 2021) [18]	*	*	*	-	*	*	*	*	*	********
(May, Barreto et al. 2021) [19]	*	*	*	-	*	*	-	*	*	*******
(Abdeljaleel et al. 2022) [20]	*	*	*	-	*	*	*	*	*	********
(Nowshad, Alam et al. 2010) [21]	*	*	*	-	*	*	*	*	*	********
(Rainone, Merlino et al. 2013) [22]	*	*	*	-	*	*	*	*	*	********
(Salahudeen, Doshi et al. 2013) [23]	*	*	*	-	*	*	*	*	*	********
(Shen, Wang et al. 2020) [24]	*	*	*	-	*	*	*	*	*	********
He Xiaoqin [25]	*	-	*	-	-	-	*	*	*	*****
Li Yuejia [26]	*	*	*	-	*	*	*	*	*	********
Liu Shuiying, et al. [27]	*	*	*	-	*	*	*	*	*	********
Peng Zhangjing [28]	*	*	*	-	*	*	*	*	*	********

^*^indicates criterion met,—indicates significant of criterion not met

### Methodological quality assessment

All included studies were of medium to high quality, as shown in Table 3 for methodological quality assessment.

## Main results: incidence of AKI

### Incidence of AKI in critically Ill patients with malignancy

**Fig. 2:**
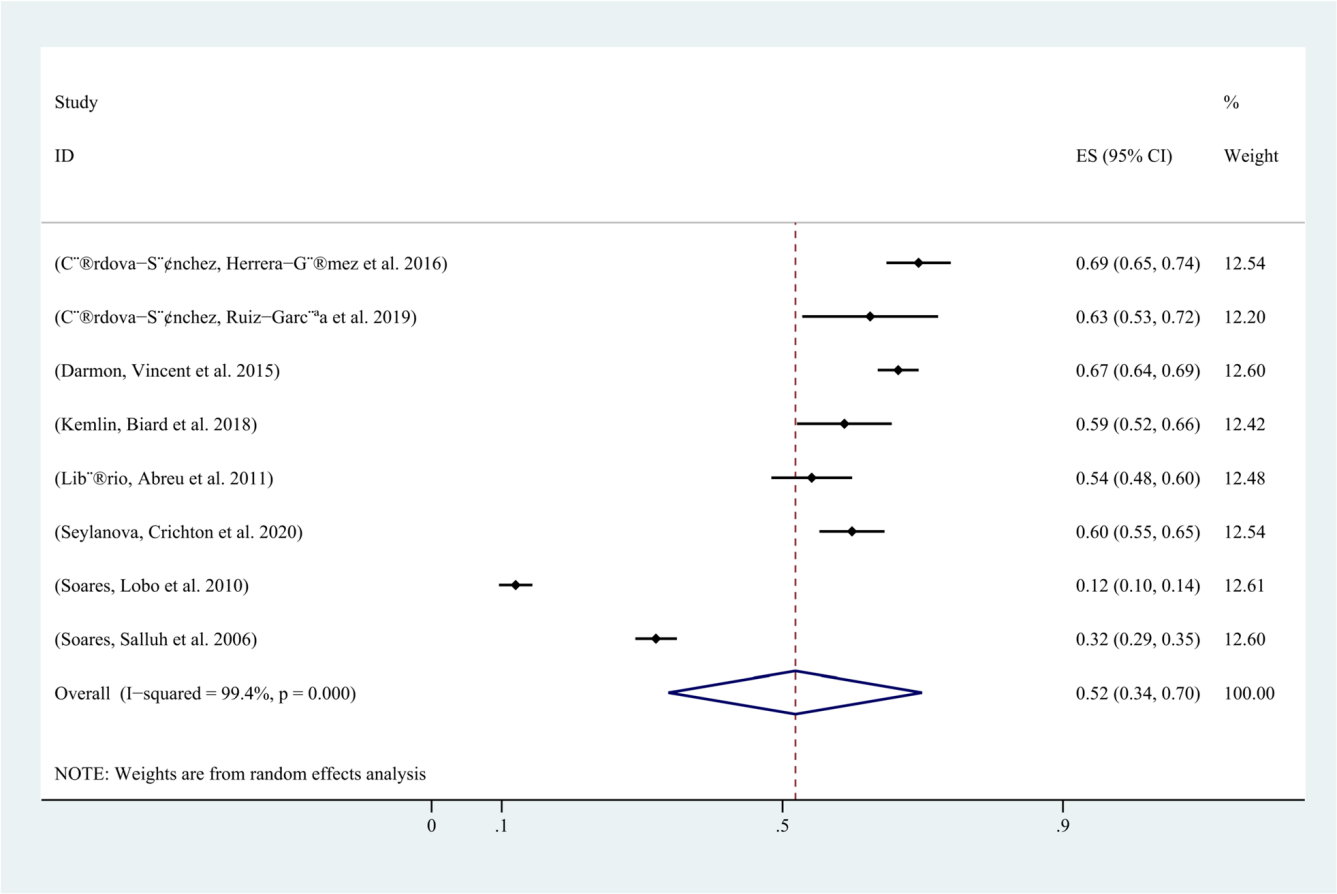
Forest plot showing the incidence of AKI in critically ill patients with malignancy

**Fig. 3:**
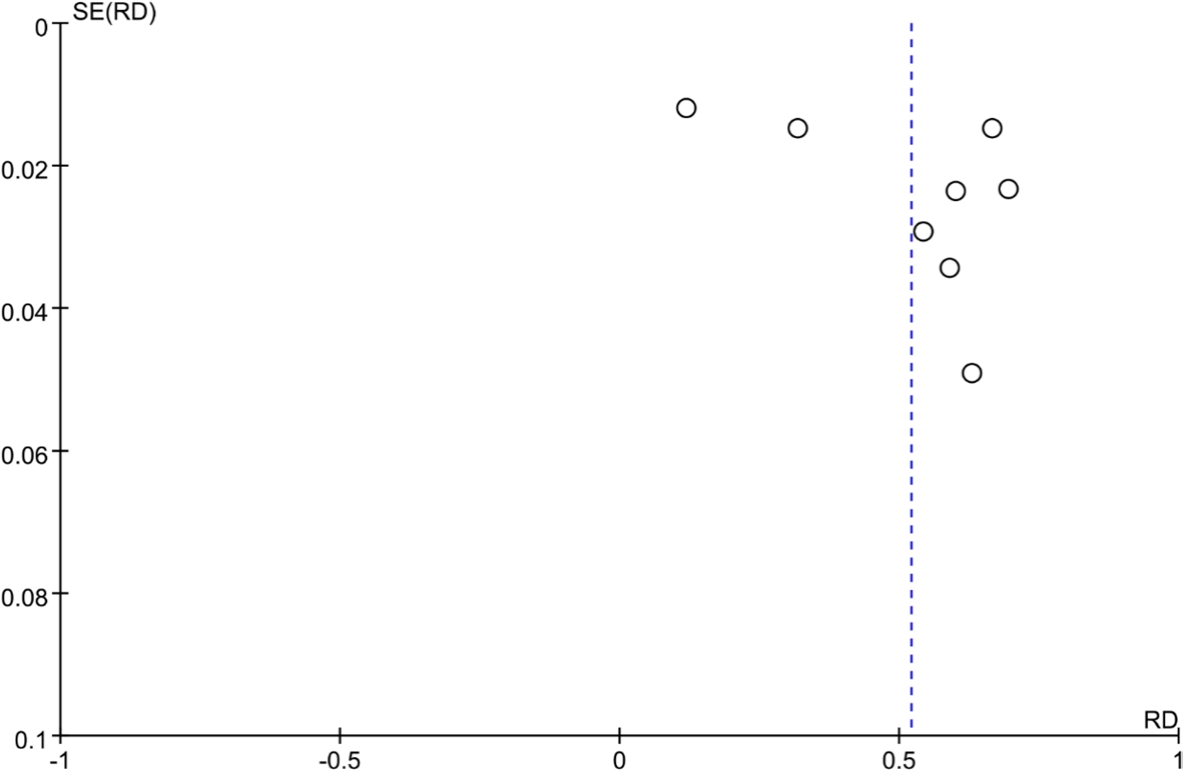
Funnel plot of publication bias in the incidence of AKI in critically ill patients with malignancy

**Fig. 4:**
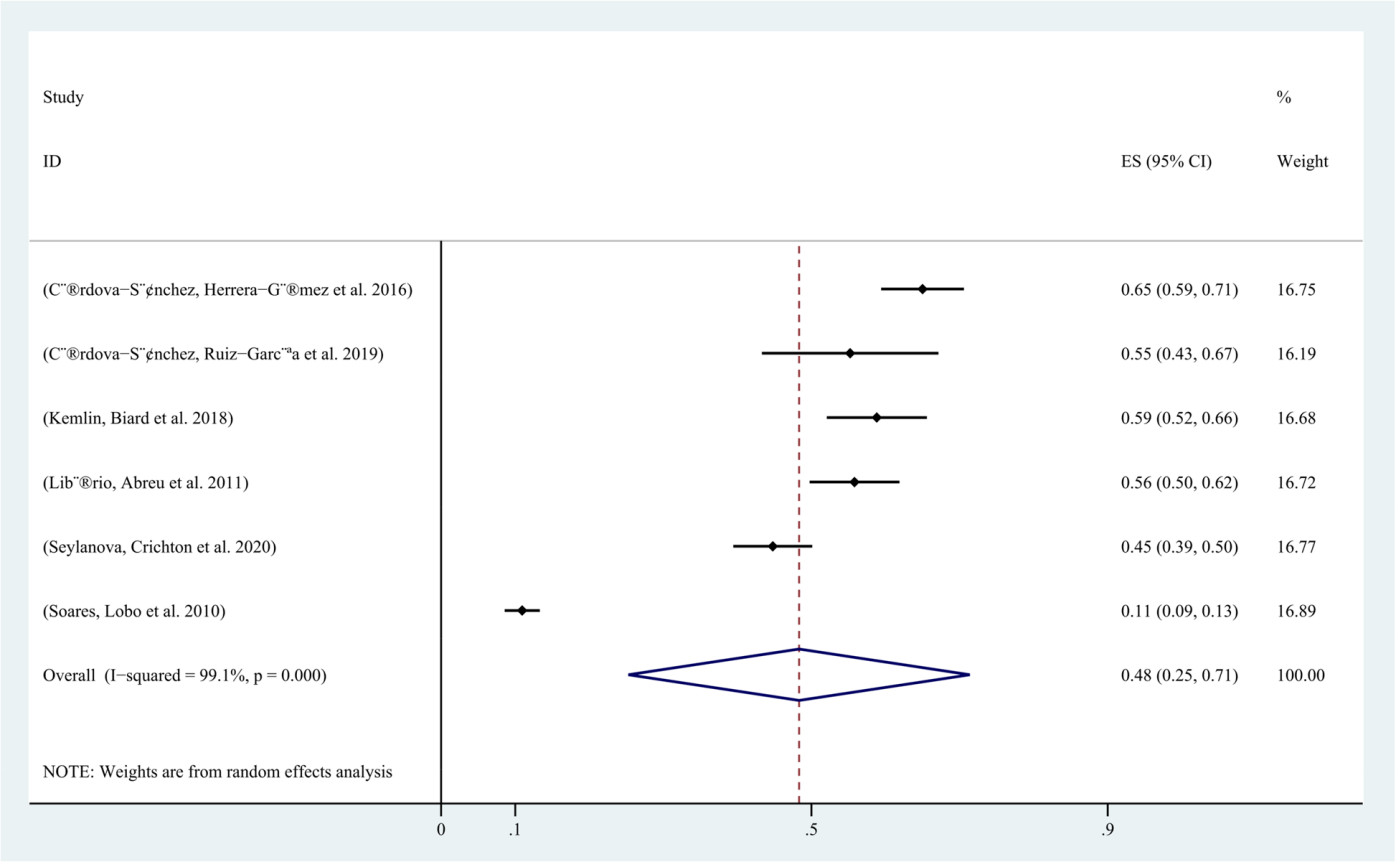
Forest plot showing subgroup analysis of the occurrence of AKI in patients with solid tumors with severe disease

**Fig. 5:**
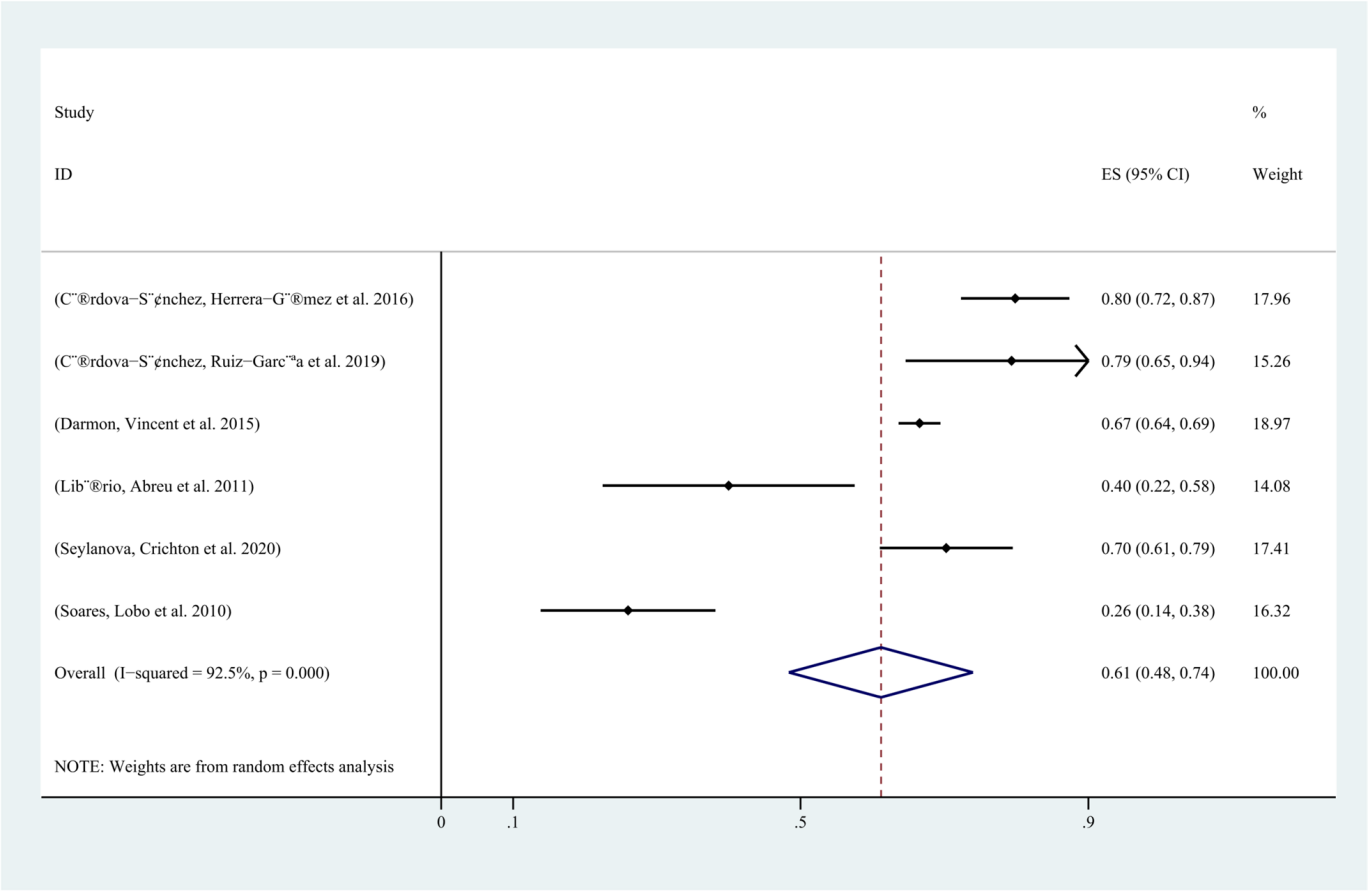
Forest plot showing the subgroup analysis of the incidence of AKI in critically ill patients with hematologic malignancies

In all 8 included studies, the incidence of AKI in critically ill cancer patients was reported. Except for 6 studies that did not use the KDIGO guideline for AKI definition, all other studies defined AKI according to the KDIGO guideline. Among the 4107 included patients, 1932 developed AKI, and the incidence of AKI in critically ill cancer patients ranged from 12% to 69.4%. The pooled incidence of AKI in critically ill patients with malignancy was 52% (95% CI, 34%-70%; I2 = 99%) (Fig. 2). The funnel plot showed significant heterogeneity among studies (Fig. 3), which could not be attributed to a specific factor through sensitivity analysis. Therefore, subgroup analysis was performed based on the type of cancer, revealing that the incidence of AKI was 48% (95% CI, 25%-71%; I2 = 99%) in patients with solid tumors (Fig. 4), and 61% (95% CI, 48%-74%; I2 = 93%) in patients with hematological malignancies (Fig. 5).

### Incidence of AKI in patients with malignancy

**Fig. 6:**
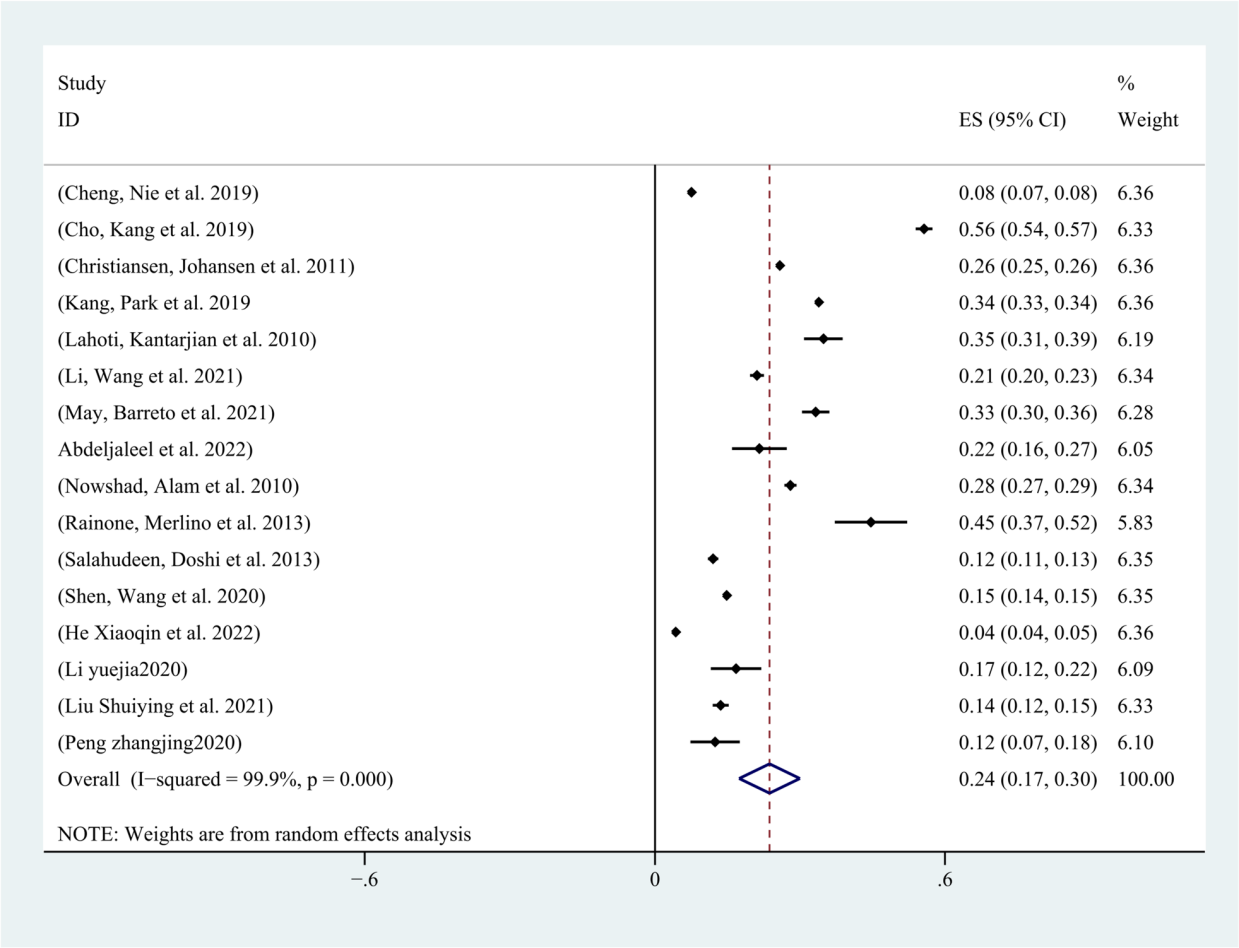
Forest plot showing the incidence of AKI in patients with malignant tumors

**Fig. 7:**
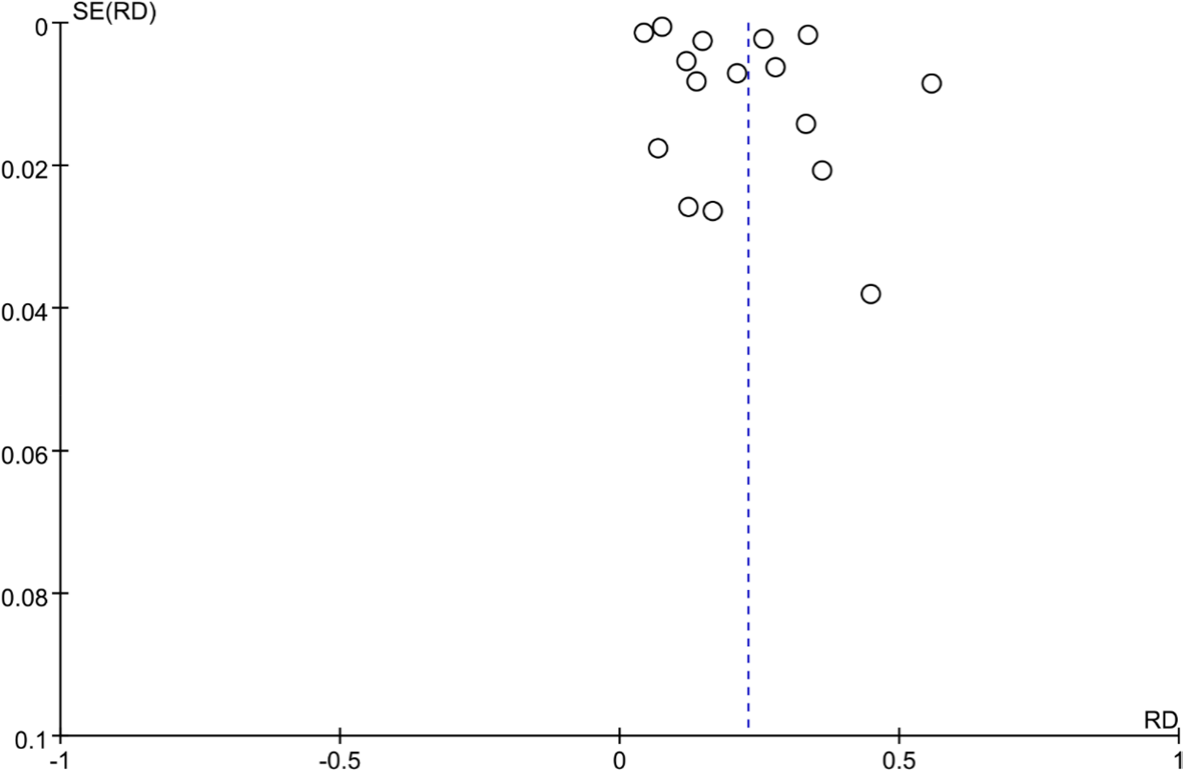
Funnel plot of publication bias in the incidence of AKI in patients with malignancy

**Fig. 8:**
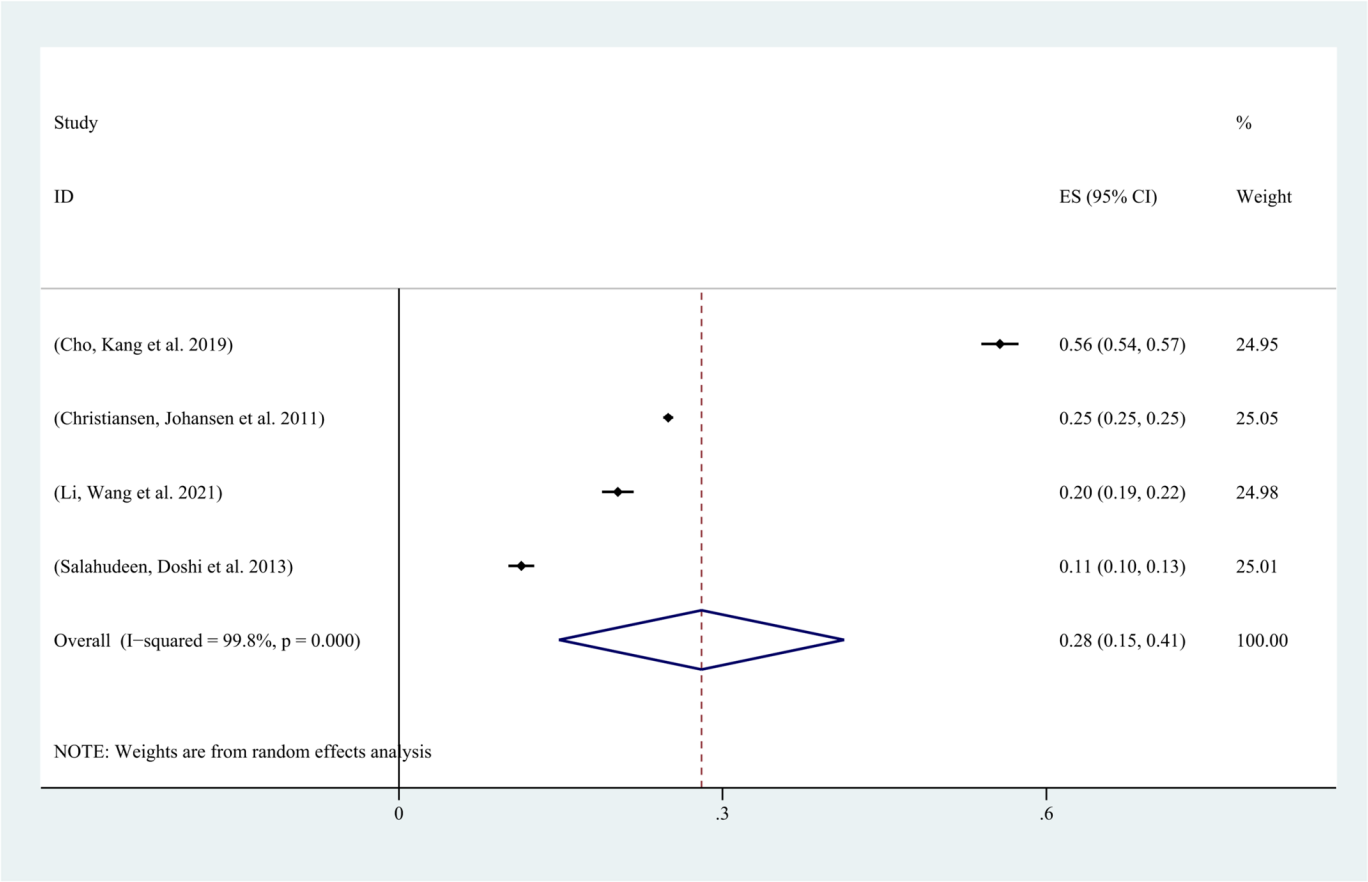
Forest plot showing subgroup analysis of the occurrence of AKI in patients with solid tumors

**Fig. 9:**
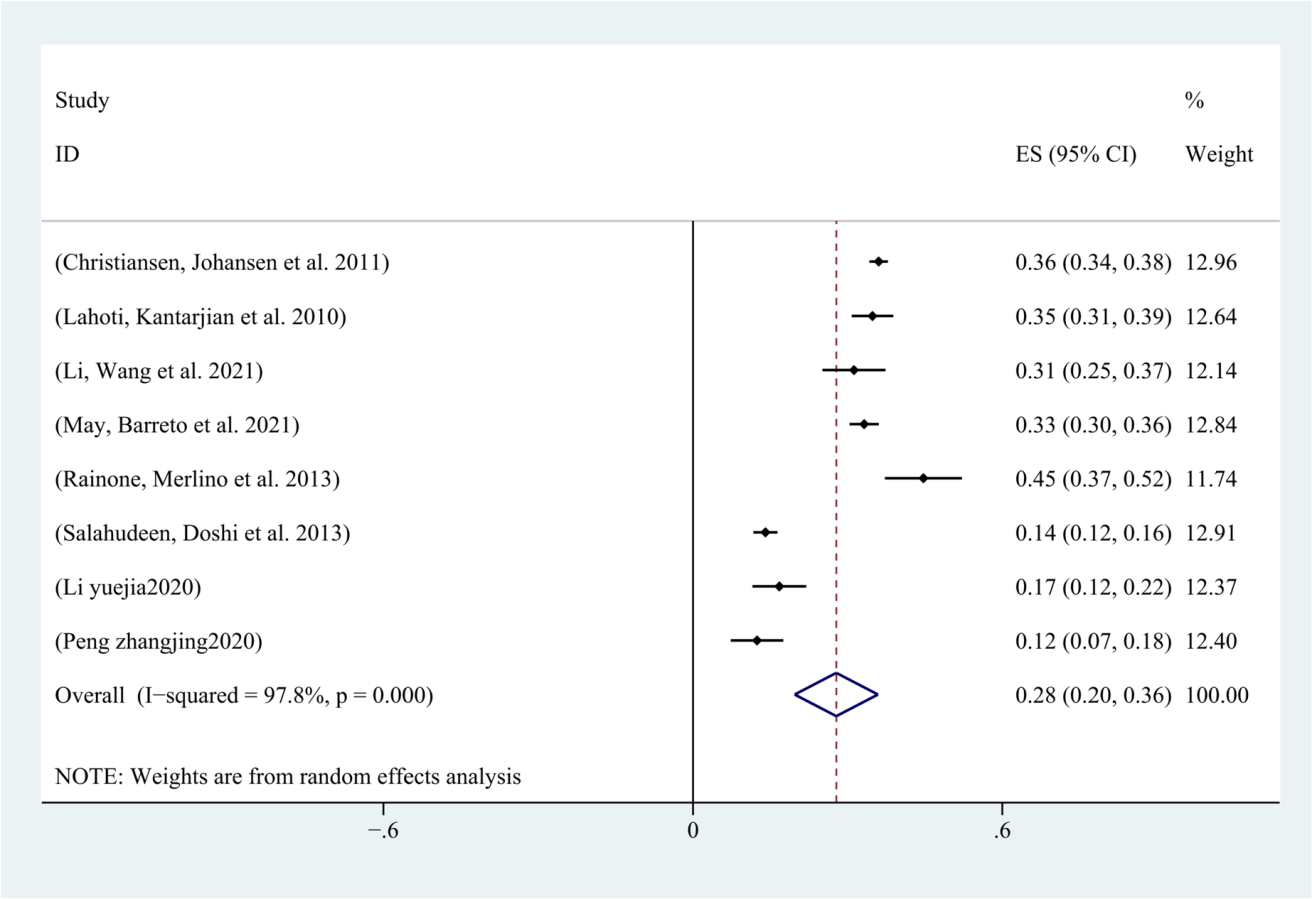
Forest plot showing subgroup analysis of the incidence of AKI in patients with hematologic malignancies

All 16 included studies reported the incidence of AKI in patients with malignancy. Except for four studies that did not use the KDIGO guideline for AKI definition, all other studies defined AKI according to the KDIGO guideline. Among the 292,874 included patients, 51,211 developed AKI, and the incidence of AKI in patients with malignancy ranged from 4.26% to 45%. The pooled incidence of AKI in patients with malignancy was 23% (95% CI, 17%-29%; I2 = 100%) (Fig. 6). The funnel plot was approximately symmetrical (Fig. 7), but showed significant heterogeneity among studies, which could not be attributed to a specific factor through sensitivity analysis. On the left side of the graph, where points aggregate more densely, it suggests that smaller studies with larger standard errors may be showing a broader range of effect sizes. This could indicate potential publication bias or other sources of heterogeneity. On the right side, where points are relatively dispersed, larger studies with smaller standard errors are contributing to a narrower range of effect sizes. This could signify greater precision and reliability in those studies.Therefore, subgroup analysis was performed based on the type of cancer, revealing that the incidence of AKI was 28% (95% CI, 15%-41%; I2 = 100%) in patients with solid tumors (Fig. 8), and 28% (95% CI, 20%-36%; I2 = 98%) in patients with hematological malignancies (Fig. 9).

## The following are the secondary results of the study

In 4 studies, 5 reports identified risk factors for AKI in critically ill patients with malignant tumors, as shown in Fig. 1, including age, history of hypertension, tumor lysis syndrome, exposure to nephrotoxic drugs, multiple myeloma, sepsis, hypovolemia, outflow obstruction, simplified acute physiology score, pelvic cancer, recent nephrotoxic chemotherapy within the last 3 months, low hemoglobin, poor renal function, and high APACHE II, SOFA, and SAPS II scores. Due to the inability to quantitatively synthesize these results, a qualitative analysis was conducted.

In 11 studies, 20 reports identified risk factors for AKI in critically ill patients with malignant tumors, as shown in Fig. 2, including age, male gender, elevated baseline serum creatinine, shock, urinary obstruction, comorbid diabetes, hypertension, low serum albumin and hemoglobin, use of vancomycin, diuretics, liposomal amphotericin B, vasopressors, leukopenia, hypoalbuminemia, chemotherapy, congestive heart failure, chronic kidney disease, antidiabetic and antibiotic medication, hyperglycemia, stem cell transplantation, malignant hematologic disorders, hypercalcemia, hyperphosphatemia, proteinuria, higher percentage of plasma cells in the bone marrow, contrast media, hyponatremia, hypomagnesemia, higher WBC count, and use of NSAIDs and diuretics. Due to the inability to quantitatively synthesize these results, a qualitative analysis was conducted.

## Discussion

Acute kidney injury (AKI) is the main cause of poor prognosis in patients. Approximately 13.3 million people develop AKI each year, with 85% living in developing countries. Although there is currently no evidence of a direct link between AKI and death, about 1.7 million people die from AKI each year [29].

In this systematic review and meta-analysis, we found that the incidence of AKI is high in patients with malignant tumors, especially in critically ill patients with malignant tumors. Independent risk factors for AKI in critically ill patients with malignant tumors include low blood volume, sepsis, outflow tract obstruction, and SOFA score.

In the first multicenter cross-sectional study of AKI epidemiology in ICU patients using the complete KDIGO criteria, AKI occurred in 52.3% of ICU patients. Adjusted risks and mortality rates for AKI were similar across different continents and regions, with sepsis and low blood volume being the most common causes of AKI [30]. In this study, the incidence of AKI in malignant tumors was 52% (95% CI 34–70%, I2 = 99%) in the ICU, which is consistent with the incidence in other patients. However, one multicenter study by Soares, Lobo et al. [11] in Pakistan had a much lower incidence of AKI than other studies, and excluding this study showed that the incidence of AKI in critically ill cancer patients was higher than that in other critically ill patients. Tumor may be a risk factor for AKI in critically ill patients. The incidence of AKI in patients with hematological malignancies is higher than that in patients with solid tumors (48% vs. 61%), and hematological malignancies lead to a higher incidence of AKI in critically ill patients. Independent risk factors for AKI are sepsis and low blood volume. Compared with non-tumor AKI, the in-hospital mortality rate of tumor AKI is higher [2].

The kidney is an important organ with excellent ability to regulate blood flow and is easily affected by poor organ perfusion. Therefore, ensuring renal perfusion is necessary and prioritized for preventing AKI in cancer patients [31]. In this study, the incidence of AKI in all cancer patients was 23% (95% CI 17–29%), and the incidence of AKI in patients with solid tumors was consistent with that in patients with hematological malignancies. This result may be due to the small number of solid tumors included in the literature and the small sample size of hematological malignancies. Compared with the global meta-analysis of AKI incidence [3], the incidence of AKI in cancer patients is slightly higher, with different risk factors including chemotherapy, tumor metastasis, and tumor lysis syndrome. Therefore, cancer increases the incidence of AKI in patients, and the use of antibiotics, such as vancomycin, is a risk factor for AKI in cancer patients, so infection control should be strengthened.

The greatest advantage of this study is that it is the first comprehensive overview and combined analysis of the incidence and risk factors of AKI in cancer patients to date, and includes a comparison between critically ill and non-critically ill patients, demonstrating the importance of hemodynamics in critically ill patients. Additionally, the study has a large and high-quality sample size, thus providing a certain degree of credibility. However, the study also has some limitations, including the fact that all the included studies were observational and lacked control groups, leading to significant heterogeneity in the results. There was also heterogeneity observed in the subgroup analysis of different types of cancer. The sample size of critically ill cancer patients with AKI was small, and the inclusion of studies on malignant tumors made it difficult to accurately compare the incidence and risk factors of AKI in critically ill and non-critically ill patients. Moreover, the study only differentiated between solid tumors and hematological tumors, and further research is needed to distinguish the incidence rates and specific risk factors of each tumor type.

## Conclusion

The incidence of acute kidney injury (AKI) in cancer patients with critical illness aligns with rates observed in other critically ill cohorts. Independent risk factors contributing to AKI comprise sepsis and hypovolemia. Specifically, within the broader patient population, the occurrence of AKI is slightly elevated in cancer patients compared to their non-cancer counterparts, indicating cancer as a discernible risk factor for AKI.

### Supplementary Information

**Additional file 1.** Research methods and reporting.

## Data Availability

All data generated or analyzed during this study are included in this published article.
